# Precision Medicine in Alzheimer’s Disease (AD): Observed Efficacy Treatment Effects from Blarcamesine Clinical AD Trials

**DOI:** 10.1002/alz70859_106527

**Published:** 2025-12-26

**Authors:** Marwan N. Sabbagh, Audrey Gabelle, Timo Grimmer, Luca Villa, Elizabeth Gordon, Mathilde Borrot, Ayoub Gueddou, Nicolas Guizard, Olivier Courreges, Kun Jin, William R. Chezem, Juan Carlos Lopez‐Talavera, Christopher U Missling

**Affiliations:** ^1^ Barrow Neurological Institute, Phoenix, AZ USA; ^2^ Montpellier Excellence University, Montpellier France; ^3^ Technical University of Munich, School of Medicine and Health, TUM University Hospital, Center for Cognitive Disorders, Munich, Bavaria Germany; ^4^ Qynapse, Paris, Île‐de‐France France; ^5^ Anavex Life Sciences Corp., New York, NY USA; ^6^ Anavex Life Sciences Corp, New York, NY USA

## Abstract

**Background:**

Precision Medicine represents a transformative approach in treating Alzheimer’s disease (AD), focusing on tailoring interventions based on individual genetic, molecular, clinical and environmental profiles in addition to confirm clear Mechanism of Action (MoA). This strategy aims to optimize treatment outcomes by identifying patient subgroups that are most likely to benefit or benefit at a higher degree from specific therapies, thus reducing inefficacies and potentially adverse effects associated with generalized treatment regimens.

**Method:**

Blarcamesine, a selective modulator of the Sigma‐1 receptor (SIGMAR1), has garnered attention due to its role in autophagy, cellular stress response, protein homeostasis, and neuroprotection. Recent clinical studies have confirmed its effects in the majority of AD patients who carry the homozygous wild‐type (WT) SIGMAR1 genotype (∼70% of the whole population) (Hampel et al., 2020).

**Result:**

In the Phase IIb/III ANAVEX2‐73‐AD‐004 trial, a clear MoA of oral once daily blarcamesine, a pre‐specified efficacy analysis confirmed the importance of SIGMAR1 to clinical response. While all trial participants improved significantly versus placebo after 48 Weeks, 36.3% for ADAS‐Cog13 and 27.6% for CDR‐SB, respectively, a further, also significant, improvement was observed in the prespecified analysis of SIGMAR1 carriers. Compared to the ITT group, participants carrying the SIGMAR1 WT gene observed further improvement versus placebo of 49.8% and 33.7% for the primary and secondary key endpoints ADAS‐Cog13 and CDR‐SB, respectively (Macfarlane et al., 2025).

**Conclusion:**

Thus, the specificity of blarcamesine to this genotype underscores the importance of understanding clear MoA and potential for further improved personalized medicine in AD treatment, as therapies targeting individual molecular profiles could achieve more substantial and sustained benefits. While Precision Medicine holds great promise for improving the treatment paradigm of AD by offering more personalized, likely more effective therapies, it also introduces challenges related to potential cost, data privacy, ethical concerns, and inequities in access. Addressing these issues is crucial to ensure that the benefits of Precision Medicine can be realized for all patients.

